# 3D-GIoU: 3D Generalized Intersection over Union for Object Detection in Point Cloud

**DOI:** 10.3390/s19194093

**Published:** 2019-09-22

**Authors:** Jun Xu, Yanxin Ma, Songhua He, Jiahua Zhu

**Affiliations:** 1 College of Information Science and Engineering, Hunan University, Changsha 410082, China; 2 College of Meteorology and Oceanography, National University of Defense Technology, Changsha 410073, China

**Keywords:** 3D object detection, point cloud, voxel, convolutional neural networks, 3D generalized intersection over union

## Abstract

Three-dimensional (3D) object detection is an important research in 3D computer vision with significant applications in many fields, such as automatic driving, robotics, and human–computer interaction. However, the low precision is an urgent problem in the field of 3D object detection. To solve it, we present a framework for 3D object detection in point cloud. To be specific, a designed Backbone Network is used to make fusion of low-level features and high-level features, which makes full use of various information advantages. Moreover, the two-dimensional (2D) Generalized Intersection over Union is extended to 3D use as part of the loss function in our framework. Empirical experiments of Car, Cyclist, and Pedestrian detection have been conducted respectively on the KITTI benchmark. Experimental results with average precision (AP) have shown the effectiveness of the proposed network.

## 1. Introduction

The task of object detection is to find the objects of interest in a given scene and determine their category and location. In the past few years, deep learning has made substantial progress in many fields due to its powerful feature learning ability, ranging from object recognition [1,2] to natural language processing [3,4]. Deep learning-based object detection methods have achieved a really high detection accuracy and are widely used in various practical applications, such as autonomous driving [5,6,7,8,9], mobile robots [10,11], video surveillance [12,13,14], and so forth.

There are some three-dimensional (3D) object detection techniques based on RGB images [6,7]. However, due to the loss of sophisticated spatial structure information in the process of projecting a 3D scene into a two-dimensional (2D) image, the performance of 3D object detection in RGB image is poor. As a result, this is limited for autonomous driving and robot vision, since these applications pay more attention to the 3D positional information of an object. Compared with RGB images, point cloud contains 3D spatial and structural information of the given scene. In addition, with the rapid development of LIDAR, the acquisition of point cloud is becoming more and more convenient. Therefore, point cloud-based 3D object detection has become an important component in many 3D applications.

Currently, deep learning-based 3D object detection in point cloud algorithms has a main challenge: the low detection precision. To solve this problem, some technologies [5,15] use a 2D detection algorithm in an image to locate the object, then use bounding box regression to achieve 3D object detection. According to the detection results of a KITTI data set [16], they have achieved good results thanks to the accurate 2D detection in images. However, these methods have two problems: they are highly dependent on 2D object detection technology and have an expensive time cost. To solve these problems, we propose our solutions: 1. Only point cloud is used for 3D object detection to reduce the time cost, 2. Feature maps of different layers are aggregated to improve the expressive ability of features, 3. A new loss function, 3D Generalized Intersection over Union (GIoU) is proposed to optimize the alignment of 3D prediction and ground truth bounding box, so as to improve the precision of 3D object detection.

In the 3D object detection network, as the depth of the network increases, the size and receptive field of the feature map also change. The lower-level feature map has high resolution and contains smaller receptive field and more detailed texture information. The high-level feature map has low resolution and contains larger receptive field and more semantic information. The integration of different levels of feature maps can improve the comprehensive expression ability of feature maps. Based on this observation, this paper proposes a Backbone Network, in which the low-resolution feature map is spliced with the high-resolution feature map after the up-sampling. Features with stronger expression ability are obtained after the fusion of features of different levels.

As we know, object detection is a multitask learning problem, which consists of object classification and object localization. Actually, bounding box regression plays an important role in object localization. Many superior object detectors rely on accurate bounding box regression to locate objects, such as VoxelNet [17], SECOND [18], and Complex-YOLO [19]. Although optimizing the architecture of deep neural network is a trend to improve the accuracy of bounding box, designing a reasonable regression loss function is also an important way. Consequently, various regression loss functions have been proposed. Among the current popular object detectors, the $l_{1}$-smooth and $l_{2}$-norm are the mainstream of loss functions used to optimize bounding box, where the $l_{2}$-norm is also known as the mean square error (MSE). Nevertheless, these functions cannot represent the core evaluation criteria (i.e., Intersection over Union (IoU)). However, there is a problem with the IoU as a loss function, that is, when the two bounding boxes are completely out of coincidence, optimization cannot be performed. In addition, IoU cannot reflect the alignment of two bounding boxes. To solve these problems, Hamid et al. [20] proposed 2D Generalized Intersection over Union (GIoU) for 2D object detection, which solved the problem of unification of loss function and evaluation criteria and improved the accuracy of 2D object detection.

However, one of the drawbacks of 2D GIoU is that it only applies to 2D object detection. To break this dilemma, this paper proposes a 3D GIoU regression loss function for 3D object detection. The ablation experiments show that the use of 3D GIoU can actually improve the detection performance.

In particular, the main contributions of this paper can be summarized as follows:

A Backbone Network is designed to aggregate the low-level features and high-level features for 3D object detection, which improves the performance of detection and enhances the robustness of the network.

3D GIoU loss function is proposed to optimize bounding box regression.

The proposed detection pipeline is evaluated on a KITTI benchmark dataset, which demonstrates that the proposed network is superior to other algorithms in average precision (AP).

## 2. Related Work

3D object detection methods can be divided into three categories by the representations of input data, therefore, monocular image-based, point cloud-based, and multimodal fusion-based methods.

### 2.1. Monocular Image-Based Detection

Monocular image-based 3D detection is the most challenging for the lack of 3D space information, but there are still some studies [6,7] that have focused on it, mainly for two reasons. On the one hand, it is a fundamental problem with great impact; on the other hand, the equipment for obtaining monocular image is more convenient and much cheaper. Considering the fact that the objects should be on the ground plane, to obtain 3D proposal from monocular image, Mono3d [6] exhaustively obtained 3D proposals from several predefined 3D regions. To select the best candidates, each candidate box is scored via encoding several intuitive potentials, such as contextual information, typical object shape, size, and location priors. The expensive computing cost of 3D sliding windows in Mono3d [6] brings a serious problem of inefficiency. To solve this problem, GS3D [7] first predicted the position, category, and orientation of 2D bounding box in a monocular image. Then it estimated the size of 3D box and roughly calculated the position of 3D candidate box in camera coordinates. The resulting 3D bounding box is projected as a front view (FV), a bird’s eye view (BEV), and a side view. Next, the 3D structural features extracted from projected surface regions and the texture information extracted from 2D box were merged. Finally, to improve the performance of detection, the fused features were used to refine the 3D bounding box. Compared with other monocular image-based 3D object detection methods, GS3D [7] achieves superior performance. However, the detection accuracy is far from meeting the requirements of automatic driving and other applications.

### 2.2. Point Cloud-Based Detection

Compared with monocular images, point cloud is regarded as an important information source for 3D object detection, since it directly reflects the real-world scenes. However, due to 3D point cloud being irregular, deep learning cannot be directly applied to object detection in point cloud. There are two popular methods to transform point cloud to regular data, and then input the transformed data to a 3D object detection network. The first method is projecting a point cloud to 2D plane to achieve 2D images [19,21,22]. To utilize the 3D data more efficiently, Complex YOLO [19] and PIXOR [21] projected point cloud to a BEV and applied 2D detection on the projected image. Although the processing method of the projection was efficient, it lost the spatial structure information of the point cloud, which led to poor detection performance.

Instead of projecting point cloud to 2D planes, an alternative method is transforming point cloud to 3D voxel grids, and then processing them with 3D convolution [17,18,23]. For the VoxelNet [17] and SECOND [18], both are one-stage detectors, a simplified PointNet [24] is applied to each non-empty voxel for extracting voxel-wise feature. After that, the entire point cloud is represented as a four-dimensional (4D) tensor. Then the 4D tensor is processed by a series of 3D convolutional layers, followed by region proposal network (RPN) [25], where RPN is used to predict the classification score and the bounding box regression map. In this paper, we use voxelization to convert point clouds into regular data for 3D object detection.

### 2.3. Multimodal Fusion-Based Detection

Several 3D object detection techniques [26,27,28,29,30,31] used a combination of RGB image and depth map. For example, Liu et al. [27] used convolutional neural networks (CNN) to extract color features from RGB image, and used convolutional deep belief networks (CDBN) to extract geometric features from depth map. Then, advanced visual features and geometric features were extracted with deep belief networks (DBN). Next, the learned features were fused to obtain a 3D multimodality feature for object detection. Deng et al. [28] used CNN to extract the appearance and geometric features from RGB and depth images respectively, and obtained 2D detection results from RGB image. Then these 2D bounding boxes were combined with geometric features, and classification results were converted into 3D space. Finally, the bounding box regression was used to refine these 3D boxes. Slightly different from [28], Luo et al. [29] concatenated the appearance and geometric features directly, and then the concatenated features were used for determining the final detection results. These methods have a large amount of computation cost, which leads to a slow detection speed, since these methods used different branches to extract the appearance features and geometric features, respectively.

In addition, some methods [5,15,32,33,34] fused RGB image and point cloud for 3D object detection. Typically, MV3D [5] converted point cloud into a BEV representation. To obtain more scene information, a BEV and FV of point cloud were fed into the detection network with RGB image. 3D candidate proposals were obtained from BEV of point cloud with CNN, since BEV suffers less occlusion. Then, candidate proposals were projected to FV and RGB image. Finally, features learned from the three 2D views were fused for object classification and bounding box regression. F-PointNet [15] is a two-stage 3D object detector that used RGB image to detect in the first stage, resulting in 2D detection boxes. In the second stage, the 2D detection results were projected into point cloud to form point cloud frustum, which was segmented by PointNet [24]. Finally, the 3D bounding boxes were calculated. Obviously, the prediction of 3D bounding box in these studies relies heavily on the 2D region proposal network, although they have achieved better detection performance. Different from these approaches, we only use point cloud data to achieve higher accuracy of 3D object detection.

## 3. Method

**Figure 1:**
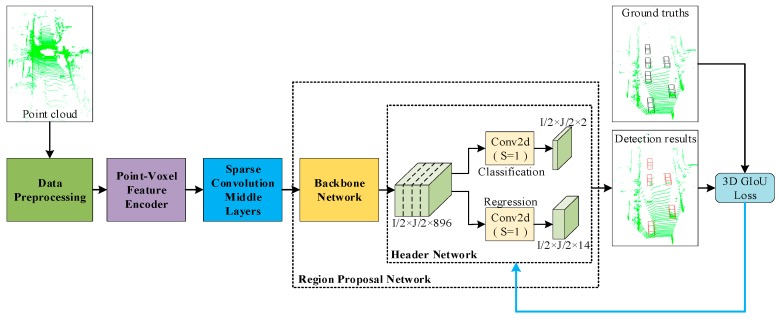
Three-Dimensional Generalized Intersection over Union (3D-GIoU) Architecture. The network takes point cloud as input. After the point cloud is discretized into 3D voxel grids, Point-Voxel Feature Encoder is used to learn voxel-wise features. Then, these features are processed by Sparse Convolution Middle Layers and sent to the Region Proposal Network to predict the classification score and the bounding box regression map. Last, the detection results and ground truth bounding boxes are used to calculate 3D GIoU loss, and 3D GIoU loss is used for optimizing the bounding box regression.

In this section, the network proposed is introduced, which is named 3D-GIoU. The whole detection network is shown in Figure 1, which includes four main components: (1) Data Preprocessing; (2) Point-Voxel Feature Encoder; (3) Sparse Convolution Middle Layers; (4) Region Proposal Network, which consist of Backbone Network and Header Network.

### 3.1. Data Preprocessing

**Figure 2:**
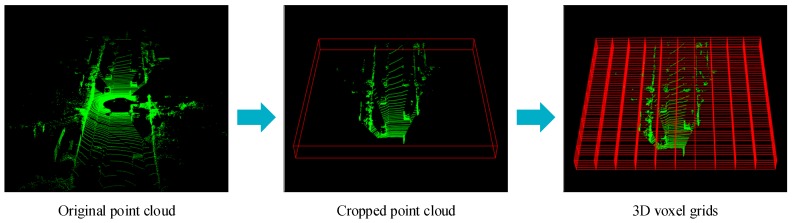
Voxelization of point cloud. Firstly, the original point cloud is cropped according to the fixed size $L\times W\times {H\;m}^{3}$, and then the cropped point cloud is further transformed into 3D voxel grids.

Since 3D point cloud is irregular and the data input to CNN must be regular, point cloud is firstly transformed into regular data by discretizing them into 3D voxel grids. For a given point cloud, we only deal with a point cloud scene of size $L\times W\times {H\;m}^{3}$ in the directions of X, Y and Z axes, and points beyond this range are discarded. In addition, according to the coordinate transformation matrix of the LIDAR to the left camera in KITTI benchmark [16], we remove points beyond the field of view of left camera. Then, the cropped point cloud is discretized into voxels of size $D_{x}$, $D_{y}$, and $D_{z}$ along the three coordinate axes. Therefore, a total of I×J×K voxels are obtained, where $I=L/D_{x}$, $J=W/D_{y}$, and $K=H/D_{z}$. The voxelization process of point cloud is shown in Figure 2.

A cropped 3D point cloud contains about 17,000 points and is unevenly distributed, which may bias the detection. To address this, a fixed number of points N in each voxel are obtained to alleviate the sampling deviation between voxels. Specifically, when the number of points in a voxel is greater than N, N points will be obtained by random down-sampling. On the contrary, points with all 0 are filled to the voxel when the number is less than N.

### 3.2. Point-Voxel Feature Encoder

Same to the previous work, PVFE [35] is used to obtain a voxel feature with high expressive ability. PVFE is composed of two full connection layers and one max-pooling layer. To be specific, each full connection layer is followed by Batch Normalization (BN) and Rectified Linear Unit (ReLU).

In details, points in voxel are input to PVFE in sequence. Here, we assume that there are M points (M≤N) in the voxel A, represented as point set $P=\left\{p_{1},{\;p}_{2},\;\cdots ,{\;p}_{M}\right\}\in R^{4}$. For point $p_{i}\;\left(i=1,\;2,\;\cdots ,\;M\right)$, to obtain an expressive feature, it is necessary to comprehensively consider its own information, the spatial relationship with other points $p_{j}\;\left(j\neq i\right)$, and the spatial relationship with voxel A. Therefore, the feature of each point is encoded as a (ten-dimensional) 10D vector $f=\left(x,\;y,\;z,\;r,\;x-\Delta x,\;y-\Delta y,\;z-\Delta z,\;x-x_{c},\;y-y_{c},\;z-z_{c}\right)$, where, (x, y,z) are the coordinates of $p_{i}$, r is the received reflectance, (Δx, Δy, Δz) are the mean coordinates of all unfilled points in the voxel A, and $\left(x_{c},{\;y}_{c},{\;z}_{c}\right)$ represents the center of the voxel A. Then, point features $F=\left\{f_{1},{\;f}_{2},\;\cdots ,{\;f}_{M}\right\}\in R^{10}$ in each voxel are fed into PVFE, and then a 128-D voxel-wise feature is outputted. Consequently, the whole point cloud is mapped into a 4D tensor with a shape of I×J×K×128.

### 3.3. Sparse Convolution Middle Layers

Since the output tensor of PVFE has high dimensionality, computational efficiency becomes the major problem. To improve the efficiency of 3D CNN and make a more objective comparison with SECOND [18], we inherit the sparse convolutional middle layers (SCML) of SECOND [18]. SCML is used to process the voxel-wise features learned by PVFE, it achieves small computational cost with a certain number of parameters. SCML ensures that the output spatial structure remains unchanged while improving the data processing speed, the details of SCML can be referred to in [18].

### 3.4. Region Proposal Network

**Figure 3:**
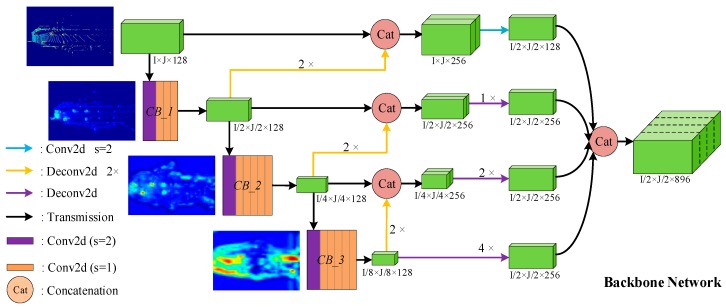
The architecture of Backbone Network. The meanings of lines and two-dimensional (2D) shapes with different colors in figure are given in the legend. Green 3D boxes represent feature maps with different sizes.

Region Proposal Network (RPN) [25] is an important part of the 3D object detection framework. The RPN proposed in this paper is composed of two parts: The Backbone Network and the Header Network. The Backbone Network consists of three components: top-bottom, bottom-top, and multiscale fusion. The structure of the Backbone Network is shown in Figure 3, where the size of tensors in the Figure 3 is marked according to the parameters of the car detection.

The top-bottom branch consists of three convolution blocks, which are named CB_1, CB_2, and CB_3 in turn. More specifically, CB_1 is composed of four convolutional layers, while CB_2 and CB_3 are both composed of six convolutional layers. Each convolution layer is followed by BN and ReLU. In addition, for car detection, the stride of the first convolution layer in CB_1, CB_2, and CB_3 is 2, and the stride of other convolution layers is 1. For cyclist and pedestrian, only the stride of the first convolutional layer in CB_2 and CB_3 is 2. The bottom-top branch consists of three deconvolution layers with a stride of 2, which are represented as three yellow lines in Figure 3. In addition, the 2D convolution of the blue line, the deconvolution layers indicated by the purple lines, and the concatenation of the last step constitute the multiscale fusion structure.

The input of the Backbone Network is a spatial feature with shape of I×J×128, which is the output of SCML. The output of the Backbone Network is a multichannel feature map with size of I/2×J/2×896, which incorporates multiscale features. Because the concatenated feature aggregates more detailed texture features and richer semantic information, the expression ability is stronger, which is important for predicting high-precision 3D bounding boxes. Finally, the output is fed into the Header Network to predict the classification score and bounding box regression map.

Different levels of feature maps examples are given in Figure 3. As shown in Figure 3, the four feature maps from top to bottom represent the input of the Backbone Network, the output of CB_1, the output of CB_2, and the output of CB_3, respectively. Obviously, the degree of abstraction of features deepens as the network hierarchy deepens, which means that the feature map contains more semantic information.

## 4. Loss Function

The loss of object detection pipeline proposed in this paper consists of three parts: (1) Classification loss; (2) bounding box regression loss; (3) 3D GIoU loss. In addition, to balance the relative importance, we add different weights to different parts. As shown in Equation (1), where $w_{1}=1$, $w_{2}=2$, $w_{3}=1$.

(1) $$L=w_{1}L_{\mathrm{cls}}+w_{2}L_{\mathrm{reg}}+w_{3}L_{3D_{\mathrm{GIoU}}\;},$$

### 4.1. Classification Loss

Since most of the bounding boxes predicted by RPN belong to negative samples, there is a large imbalance between positive samples and negative samples. This deviation makes the negative loss far greater than the positive loss during training, which is not conducive to the training of network. Therefore, the focal loss function proposed by Lin et al. [36] is adopted to obtain an effective pipeline, as shown in Equation (2).

(2) $$L_{\mathrm{cls}}=\mathrm{FL}\left(p_{t}\right)=-\alpha_{t}{\left(1-p_{t}\right)}^{r}\log\left(p_{t}\right),$$

Specifically, $p_{t}$ represents the evaluation probability value of the model for the corresponding bounding box, and the scale factor is set as $\alpha_{t}=0.25$, γ=2. Essentially, the focal loss function is a dynamically scaled cross entropy loss. When the confidence of the correct class increases, the weight ${\left(1-p_{t}\right)}^{\gamma }$ decays to zero. On the contrary, the weight increases.

### 4.2. 3D Bounding Box Regression Loss

For the bounding box regression, the 3D ground truth bounding box is parameterized as $\left(x_{g},y_{g},z_{g},l_{g},w_{g},h_{g},\theta_{g}\right)$ while the matching anchor is $\left(x_{a},y_{a},z_{a},l_{a},w_{a},h_{a},\theta_{a}\right)$, where (x,y,z) denote the center coordinate, (l,w,h) is the length, width, and height of the 3D box, and θ is the yaw rotation around Z axis. Then, we define vector $r^{*}\in R^{7}$, which encodes the regression targets. Finally, $r^{*}$ is computed as:

(3) $$\begin{array}{l} x_{r}=\frac{x_{g}-x_{a}}{d_{a}},{\;y}_{r}=\frac{y_{g}-y_{a}}{d_{a}},{\;z}_{r}=\frac{z_{g}-z_{a}}{h_{a}},\\ l_{r}=\log\left(\frac{l_{g}}{l_{a}}\right),{\;w}_{r}=\log\left(\frac{w_{g}}{w_{a}}\right),{\;h}_{r}=\log\left(\frac{h_{g}}{h_{a}}\right),\\ \theta_{r}=\theta_{g}-\theta_{a}, \end{array}$$

Then, bounding box regression loss is defined as following:

(4) $$L_{\mathrm{reg}}=\sum_{e\in \left(x,y,z,l,w,h\right)}\mathrm{SmoothL}1\left(e_{r}\right)+\mathrm{SmoothL}1\left({\sin\theta }_{r}\right),$$

### 4.3. 3D GIoU Loss

Currently, the regression loss of the bounding box (mean squared error loss, $l_{1}$-smooth loss) is the mainstream method to optimize the bounding box in the object detection. However, IoU is the most commonly used metric for comparing the similarity between two arbitrary shapes, which is also known as Jaccard index. In fact, two shapes can overlap in different ways to get the same $l_{1}$ or $l_{2}$-norms values, but when they overlap in different ways, the IoU value is different [20], which indicates that the $l_{1}$ and the $l_{2}$-norms cannot effectively reflect the detection effect. However, IoU not only reflects the distance between the predicted and ground truth bounding box, but also has scale invariance. Therefore, some object detection techniques [37,38] adopt IoU loss to optimize the bounding box. Here, given two arbitrary shapes $A,B\in R^{n}$, the IoU and IoU loss are defined follows:

(5) $$\mathrm{IoU}=\frac{\left|A\cap^{\text{​}}B\right|}{\left|A\cup^{\text{​}}B\right|},$$

(6) $${\;L}_{\mathrm{IoU}}=1-\mathrm{IoU},$$

**Figure 4:**
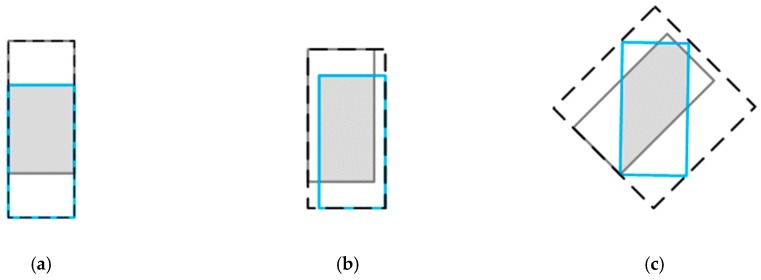
Three different ways of overlap between two rectangles with the exact same IoU values, (i.e., IoU = 0.50), but different GIoU values (i.e., from the left to right GIoU = 0.50, 0.45, and 0.09, respectively). For the case with better aligned orientation, the GIoU value will be higher.

It cannot be neglected that there are two shortcomings of IoU loss in optimizing bounding box:

(1) When the predicted and ground truth bounding box do not coincide completely, the gradient of loss function is 0, which makes it impossible to optimize;
(2) Two shapes can overlap in different ways to get the same IoU value, that is, the IoU does not reflect how overlap between two objects occurs (see Figure 4).

To solve these issues, Hamid et al. proposed a 2D GIoU loss for optimizing the bounding box in 2D object detection [20]. Motivated by [20], we propose a 3D GIoU loss function for 3D object detection, which contributes to align 3D predicted and ground truth bounding boxes.

In this paper, the optimization of the bounding box adopts two losses of $l_{1}$- smooth and 3D GIoU. In particular, $l_{1}$-smooth is firstly used to optimize all 3D bounding boxes, and then the 3D GIoU is used to optimize those bounding boxes that are judged to be positive samples. The algorithm of 3D GIoU loss is defined as Algorithm 1.


**Algorithm 1: 3D Generalized Intersection Over Union Loss**
 **Input:** The information of the predicted $B_{p}$ and ground truth $B_{g}$ bounding box:     $B_{p}=\left(x_{p},y_{p},z_{p},l_{p},w_{p},h_{p},\theta_{p}\right),\;B_{g}=\left(x_{g},y_{g},z_{g},l_{g},w_{g},h_{g},\theta_{g}\right)$
 **Output:**
$L_{3D\_\mathrm{GIoU}}$
1.Calculating projections $B_{p}^{\prime }$ and $B_{g}^{\prime }$ of box $B_{p}$ and $B_{g}$ on the bird’s eye view, respectively.$B_{p}^{\prime }=\left(x_{p}^{1},y_{p}^{1},x_{p}^{2},y_{p}^{2},\theta_{p}^{\prime }\right),\;B_{g}^{\prime }=\left(x_{g}^{1},y_{g}^{1},x_{g}^{2},y_{g}^{2},\theta_{g}^{\prime }\right)$2.$B_{c}^{\prime }=\left(x_{c}^{1},y_{c}^{1},x_{c}^{2},y_{c}^{2},\theta_{c}^{\prime }\right)\leftarrow$ the information of smallest enclosing box;3.$A_{p}\leftarrow$ the area of the 2D box $B_{p}^{\prime }$;4.$A_{g}\leftarrow$ the area of the 2D box $B_{g}^{\prime }$;5.$A_{c}\leftarrow$ the area of the 2D box $B_{c}^{\prime }$;6.$I_{2D}\leftarrow$ intersection between $B_{p}^{\prime }$ and $B_{g}^{\prime }$;7.$U_{2D}\leftarrow$ union between $B_{p}^{\prime }$ and $B_{g}^{\prime }$;8.$I_{h}\leftarrow$ the height of the intersection of $B_{p}$ and $B_{g};$9.$U_{h}\leftarrow$ the height of the union of $B_{p}$ and $B_{g};$10.$V_{p}\leftarrow$ the volume of the 3D box $B_{p}$;11.$V_{g}\leftarrow$ the volume of the 3D box $B_{g}$;12.$V_{c}\leftarrow$ the volume of the 3D box $B_{c}$, where $B_{c}$ represents the smallest 3D enclosing box;13.Calculating intersection $I_{v}$ of $B_{p}$ and $B_{g}$:**if**$I_{2D}\leq 0:$ $I_{v}=0;$**else:****if**$I_{h}\leq 0$: $I_{v}=0;$  **else:**    $I_{v}=I_{2D}\times I_{h};$14.${\mathrm{IoU}}_{3D}=\frac{I_{v}}{U_{v}}$, where $U_{v}=V_{p}+V_{g}-I_{v}$;15.${\mathrm{GIoU}}_{3D}={\mathrm{IoU}}_{3D}-\frac{\left(V_{c}-U_{v}\right)}{V_{c}}$;16.$L_{3D\_\mathrm{GIoU}}=1-{\mathrm{GIoU}}_{3D};$

**Figure 5:**
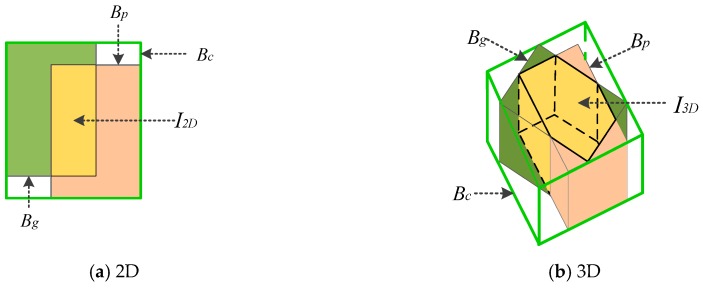
Different ways of overlap between bounding boxes in case of 2D and 3D, respectively. For (**a**) and (**b**), cyan and pink represent the predicted bounding box $B_{p}$ and ground truth $B_{g}$, respectively, and yellow represents the intersection of them. In addition, the green bounding box represents the smallest enclosing box $B_{c}$.

To better understand the calculation of the smallest enclosing box $B_{c}$ in 3D GIoU, we give 2D and 3D examples in (a) and (b) of Figure 5.

## 5. Experiments

The KITTI benchmark dataset [16] was employed to evaluate our proposed method. It contains 7481 training and 7518 testing point clouds, including three categories: car, cyclist, and pedestrian. The training dataset was divided into a training set (3712) and a validation set (3769), since the ground truth of the testing dataset is not publicly available.

### 5.1. Network Details

#### 5.1.1. Car Detection

For the car detection task, the range of point cloud taken into consideration was $L\times W\times H=\left[0,\;70.4\right]\times \left[-40,\;40\right]\times \left[-3,\;1\right]\;m^{3}$ along X, Y, and Z axis, respectively. The 3D voxel dimension was set to be $D_{x}\times D_{y}\times D_{z}=0.2\times 0.2\times 0.4{\;m}^{3}$, which led to I×J×K=352×400×10. In addition, N = 35 was set as the maximum number of points for random down-sampling within the voxel. Following the SECOND [18], the set of anchors was a 3D box with measurement of $l\times w\times h=3.9\times 1.6\times 1.56{\;m}^{3}$, which is the mean size of car and centered at z=−1m. As to the orientation, $\theta =0^{{}^{\circ}}$ or $\theta =90^{{}^{\circ}}$ was considered in our experiments.

#### 5.1.2. Cyclist and Pedestrian Detection

For cyclist and pedestrian detection, the range of point cloud was set to $L\times W\times H=\left[0,\;48\right]\times \left[-20,\;20\right]\times \left[-2.5,\;0.5\right]{\;m}^{3}$, and the size of 3D voxel was $D_{x}\times D_{y}\times D_{z}=0.2\times 0.2\times 0.3{\;m}^{3}$, which led to I×J×K=240×200×10. As with car detection, N = 35 was taken for random down-sampling of points in voxels. For the detection of cyclist, the set of anchors was a 3D box with size $l\times w\times h=1.76\times 0.6\times 1.73{\;m}^{3},$ while the size was $l\times w\times h=0.8\times 0.6\times 1.73{\;m}^{3}$ for pedestrian. Besides, all the anchors were centered at z=−0.6 m.

### 5.2. Training

In the experiments of this paper, there were only 3712 point clouds in the training set, which would inevitably lead to the overfitting of our network. To solve this problem, we introduced three different forms of data augmentation in SECOND [18]: (1) Motion; (2) global scaling and rotation; (3) sample ground truths from the database. The proposed framework was trained for 200k iterations using the Adam optimizer [39]. The initial learning rate was 0.002, the exponential decay rate was 0.8, and there was a decay every 18,750 iterations. For the detection of car, cyclist, and pedestrian, the batch size of 3 was used, distributed on a GTX 2080 Ti GPU, and the whole network took about 22 h to train.

### 5.3. Comparisons on the KITTI Validation Set

The 3D detection performance of our network on the KITTI verification set is shown in Table 1. In order to demonstrate the superior performance of the proposed detector, we compared it with other detectors, such as the MV3D [5], AVOD [33] and F-PointNet [15] which used both RGB image and point cloud, and VoxelNet [17], SECOND [18], PointPillars [40], and PVFE [35] which only used point cloud. Besides, the performance of our method for BEV object localization is given in Table 2.

Compared with 2D object detection, 3D object detection is more challenging, since it requires higher localization accuracy of 3D bounding box in space. As shown in Table 1, we can see that 3D-GIoU proposed in this paper is more suitable for 3D object detection. Specifically, for car and cyclist, 3D-GIoU significantly outperformed other approaches across all difficulty levels. Moreover, in the cyclist detection, our method achieved an AP of 63.51% in the Hard level, which is 7.72% higher than the result of SECOND [18]. In addition, in the performance for BEV object localization, which is shown in Table 2, 3D-GIoU achieved better results with respect to AP compared with other methods, although it was slightly inferior in pedestrian detection task.

**Figure 6:**
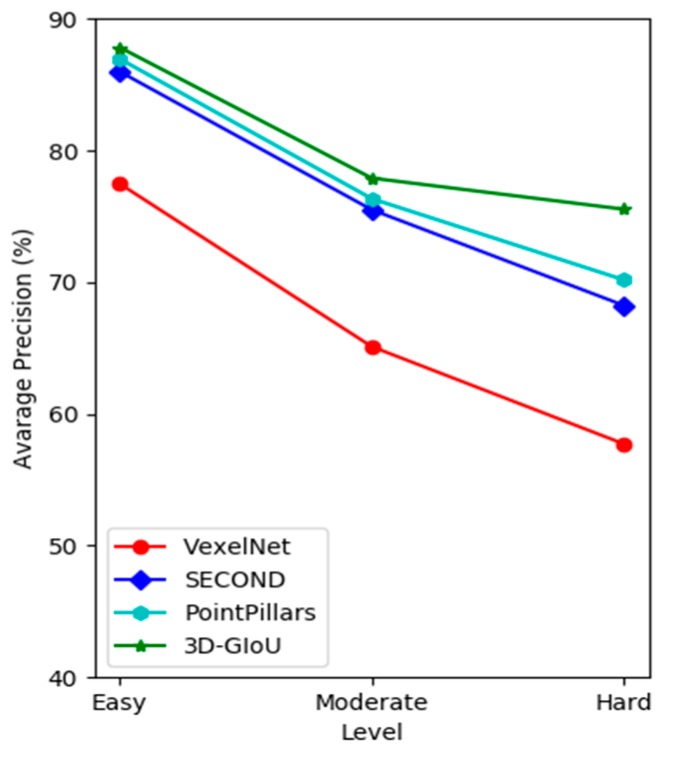
The AP of different methods on KITTI validation set with the different difficulty level (car detection).

In addition, as shown in Figure 6, it is easy to find that the AP of 3D-GIoU was significantly higher than that of other methods across three difficulty levels. In addition, the AP of 3D-GIoU decreased more slowly with the difficulty level from Easy to Moderate, then to Hard, which further demonstrates that our network has better robustness.

**Figure 7:**
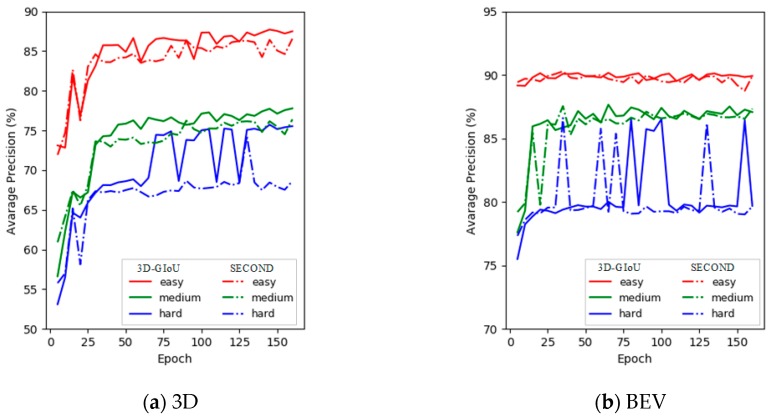
3D-GIoU vs. SECOND for the detection performance of 3D and BEV evaluation on the KITTI validation set across three difficulty levels (i.e., Easy, Moderate and Hard). In (**a**) and (**b**), the solid line represents 3D-GIoU, while the dotted line represents SECOND.

In order to compare the performance between our structure and the basic network SECOND [18] more intuitively, the training detection results on the KITTI verification set with 3D and BEV are shown in Figure 7. As shown in Figure 7, the 3D detection performance of 3D-GIoU significantly outperformed that of SECOND [18], although the BEV detection performance was not visually different from SECOND [18].

Additionally, we can see from Figure 7 that the AP of our architecture was significantly lower than SECOND [18] at the beginning of training. However, after training about 15 epochs, the performance of our network reached the level of SECOND [18] with both of 3D and BEV, and then far exceeded SECOND [18]. Obviously, the results fully demonstrate that our structure is easier to train.

### 5.4. Analysis of the Detection Results

**Figure 8:**
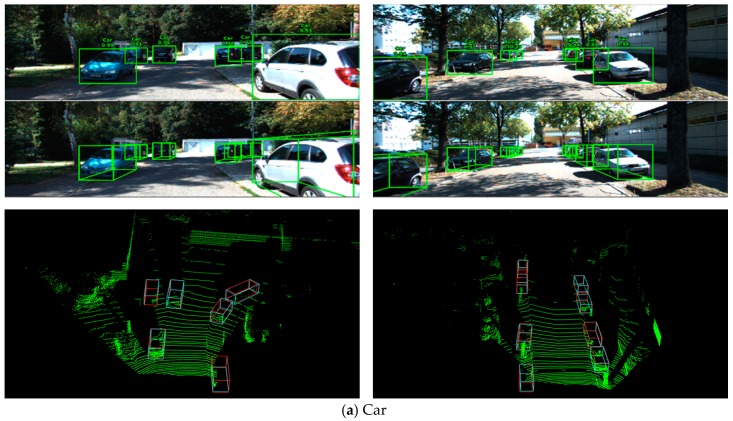

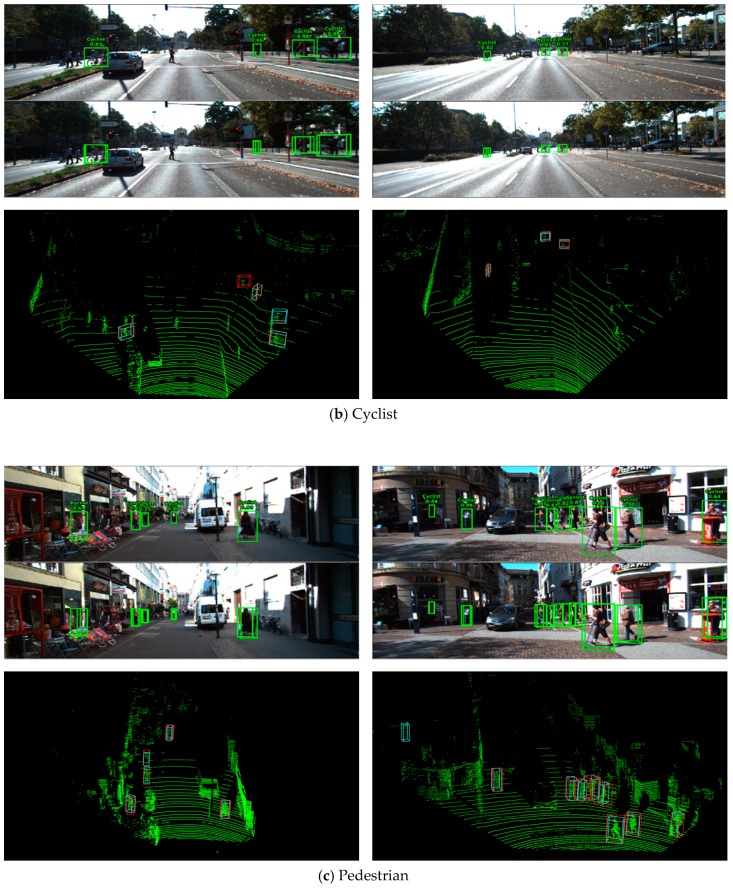
Several 3D detection results on the KITTI validation set. In each RGB image, all the 2D and 3D bounding boxes represent the detection results. The digit and word beside each 2D box represent the instance score and class. In the point clouds, teal 3D boxes indicate detection results, and 3D red boxes represent ground truths.

Some detection results on the KITTI validation set of our network are shown in Figure 8. As shown in the RGB images in Figure 6, 3D bounding boxes were also projected into corresponding images of the point cloud, resulting in 2D bounding boxes and 3D bounding boxes on the image.

#### 5.4.1. Car Detection

The four images and the associated point clouds in Figure 8a are shown as typical car detection examples. Whether it is a long- or close-distance car, our network can achieve superior detection results, even if the available points belonging to a long-distance car are few. Furthermore, the proposed network can successfully detect highly occluded cars, which is a great challenge task for other networks. Consequently, these results show that the proposed network is suitable for 3D car detection.

#### 5.4.2. Cyclist and Pedestrian Detection

The images and the associated point clouds in Figure 8b and c show the detection results of cyclists and pedestrians, respectively. It is easy to find that there were more detection errors than cars. The causes of these errors can be summarized into three points. Firstly, compared with cars, there were relatively few instances of cyclists and pedestrians in the training set, which led to insufficient training of the network. Secondly, the size of cyclists and pedestrians was smaller, that is to say, each instance contained fewer points, which made it easier to confuse with other objects with similar size. Thirdly, the positioning of the 3D bounding box of some successfully detected objects was not precise enough, which was mainly reflected in the rotation angle. Therefore, how to filter out the unrelated points, improve the object recall rate, and give more accurate 3D bounding box is a research focus of cyclist and pedestrian detection.

### 5.5. Ablation Studies

To prove the effectiveness of the 3D GIoU loss and Backbone Network proposed in this paper, we have done some ablation experiments on the KITTI validation set, and the results are summarized in Table 3. In particular, Baseline 1 represents the corrected SECOND [18], which adds the 3D GIoU loss. Correspondingly, Baseline 2 represents replacing the RPN in SECOND [18] with the RPN proposed in this article, which is composed of the Backbone Network and the Header Network.

According to Table 3, we can make the following comparison and get the corresponding conclusion:

(1) Comparing Baseline 1 with SECOND [18], it is easy to find that the proposed 3D GIoU loss can improve detection performance. In particular, the AP of the Hard level was increased by 6.4%.
(2) Comparing Baseline 2 with SECOND [18], we can find that the use of the proposed Backbone Network improved the detection performance in Hard level by 7.28%.
(3) By comparing 3D-GIoU with Baseline 1, Baseline 2, and SECOND [18], it is not difficult to find that when the 3D GIoU loss and Backbone Network are used simultaneously, the performance of 3D object detection is greatly improved.

## 6. Conclusions

In this paper, a Backbone Network and a 3D GIoU loss function are proposed for 3D object detection in point cloud. Backbone Network can effectively combine detail texture features in low-level feature maps with semantic features in high-level feature maps, and 3D GIoU loss can significantly improve the localization accuracy of 3D box. A large number of experiments have been carried out on the public KITTI benchmark, and our module has achieved excellent results, which fully demonstrate that the proposed structure is suitable for 3D object detection in point cloud.

## Figures and Tables

**Table 1:** 3D detection performance: Average precision (AP) (%) for 3D box in the KITTI valuation set.

Method	Modality	Car			Cyclist			Pedestrian		
		Easy	Mod.	Hard	Easy	Mod.	Hard	Easy	Mod.	Hard
MV3D	Img. & Lidar	71.09	62.35	55.12	N/A	N/A	N/A	N/A	N/A	N/A
AVOD	Img. & Lidar	81.94	71.88	66.38	64.00	52.18	46.61	50.80	42.81	40.88
F-PointNet	Img. & Lidar	81.20	70.39	62.19	71.96	56.77	50.39	51.21	44.89	51.21
VoxelNet	Lidar	77.47	65.11	57.73	61.22	48.36	44.37	39.48	33.69	31.50
PointPillars	Lidar	86.96	76.35	70.19	77.75	58.55	54.85	67.07	58.74	55.97
PVFE	Lidar	87.32	77.12	68.87	81.58	62.41	56.33	58.48	51.74	45.09
SECOND	Lidar	85.99	75.51	68.25	80.47	57.02	55.79	56.99	50.22	43.59
3D-GIoU	Lidar	87.83	77.91	75.55	83.32	64.69	63.51	67.23	59.58	52.69

**Table 2:** Bird’s Eye View (BEV) detection performance: AP (%) for BEV box in the KITTI valuation set.

Method	Modality	Car			Cyclist			Pedestrian		
		Easy	Mod.	Hard	Easy	Mod.	Hard	Easy	Mod.	Hard
MV3D	Img & Lidar	86.02	76.90	68.48	N/A	N/A	N/A	N/A	N/A	N/A
AVOD	Img & Lidar	88.53	83.79	77.90	68.09	57.48	50.77	58.75	51.05	47.54
F-PointNet	Img & Lidar	88.07	84.00	75.33	75.38	61.96	54.68	58.09	50.22	47.02
PIXOR	Lidar	89.38	83.70	77.97	N/A	N/A	N/A	N/A	N/A	N/A
VoxelNet	Lidar	89.35	79.26	77.39	66.07	54.76	50.55	46.13	40.74	38.11
PointPillars	Lidar	90.12	86.67	84.53	80.89	61.54	58.63	73.08	68.20	63.20
PVFE	Lidar	89.98	87.03	79.31	84.30	64.72	58.42	61.93	54.88	51.93
SECOND	Lidar	89.23	86.25	78.95	82.88	63.46	57.63	60.81	53.67	51.10
3D-GIoU	Lidar	90.16	87.92	86.55	85.35	66.91	65.06	70.16	62.57	55.52

**Table 3:** 3D and BEV detection performance: AP (%) on the KITTI valuation set.

Method	Method	Car			Cyclist			Pedestrian		
		Easy	Mod.	Hard	Easy	Mod.	Hard	Easy	Mod.	Hard
**3D**	SECOND	85.99	75.51	68.25	80.47	57.02	55.79	56.99	50.22	43.59
	Baseline 1	87.20	76.80	74.65	82.84	62.34	56.66	58.16	51.42	44.74
	Baseline 2	87.62	77.37	75.53	83.89	64.27	62.75	59.37	52.42	49.78
	3D-GIoU	87.83	77.91	75.55	83.32	64.69	63.51	67.23	59.58	52.69
BEV	SECOND	89.23	86.25	78.95	82.88	63.46	57.63	60.81	53.67	51.10
	Baseline 1	89.99	86.82	86.03	84.83	64.56	58.55	62.34	59.35	52.70
	Baseline 2	89.80	87.13	86.31	85.42	65.78	64.45	66.40	59.40	52.56
	3D-GIoU	90.16	87.92	86.55	85.35	66.91	65.06	70.16	62.57	55.52
